# Hodgkin Lymphoma in People Living with HIV

**DOI:** 10.3390/cancers13174366

**Published:** 2021-08-29

**Authors:** Jose-Tomas Navarro, José Moltó, Gustavo Tapia, Josep-Maria Ribera

**Affiliations:** 1 Department of Hematology, Institute Català d’Oncologia-Germans Trias i Pujol Hospital, Carretera de Canyet s/n, 08916 Barcelona, Spain; jribera@iconcologia.net; 2 Josep Carreras Leukaemia Research Institute, 08916 Badalona, Spain; 3 Departament de Medicina, Can Ruti Campus, Unitat Docent Germans Trias i Pujol, Universitat Autònoma de Barcelona, 08916 Badalona, Spain; jmolto@flsida.org (J.M.); gtapia.germantrias@gencat.cat (G.T.); 4 Fundació Lluita Contra la Sida, Infectious Diseases Department, Germans Trias i Pujol Hospital, 08916 Badalona, Spain; 5 Department of Pathology, Germans Trias i Pujol Hospital, 08916 Badalona, Spain

**Keywords:** HIV, hodgkin lymphoma, antiretroviral therapy, prognosis

## Abstract

**Simple Summary:**

Hodgkin lymphoma (HL) is a non-AIDS defining neoplasm, but people living with HIV (PLWH) have between a 5- and 26-fold higher risk of developing it than the general population. Epstein-Barr virus is present in almost all HIV-related HL cases, and plays an important role in its etiopathogenesis. Despite the aggressive characteristics, the prognosis of HL affecting PLWH is similar to that of the general population if patients are treated following the same recommendations. Administration of cART concomitantly with chemotherapy is highly recommended. However, this combination may be challenging due to drug–drug interactions and overlapping toxicity. Thus, interdisciplinary collaboration between hemato-oncologists and HIV specialists is crucial for the optimal treatment of both lymphoma and HIV infection.

**Abstract:**

Despite widespread use of combined antiretroviral therapy (cART) and increased life expectancy in people living with HIV (PLWH), HIV-related lymphomas (HRL) remain a leading cause of cancer morbidity and mortality for PLWH, even in patients optimally treated with cART. While the incidence of aggressive forms of non-Hodgkin lymphoma decreased after the advent of cART, incidence of Hodgkin lymphoma (HL) has increased among PLWH in recent decades. The coinfection of Epstein–Barr virus plays a crucial role in the pathogenesis of HL in the HIV setting. Currently, PLWH with HRL, including HL, are treated similarly to HIV-negative patients and, importantly, the prognosis of HL in PLWH is approaching that of the general population. In this regard, effective cART during chemotherapy is strongly recommended since it has been shown to improve survival rates in all lymphoma subtypes, including HL. As a consequence, interdisciplinary collaboration between HIV specialists and hemato-oncologists for the management of potential drug–drug interactions and overlapping toxicities between antiretroviral and antineoplastic drugs is crucial for the optimal treatment of PLWH with HL. In this article the authors review and update the epidemiological, clinical and biological aspects of HL presenting in PLWH with special emphasis on advances in prognosis and the factors that have contributed to it.

## 1. Introduction

Since the introduction of combination antiretroviral therapy (cART) the incidence of opportunistic infections and AIDS defining cancers, such as Kaposi sarcoma (KS), aggressive B-cell non-Hodgkin lymphomas (NHL) and invasive cervical cancer, has decreased in people living with HIV (PLWH) [1,2]. However, lymphoma is the most frequent AIDS-defining neoplasm in developed countries and is still one of the most frequent neoplastic causes of death among HIV-infected individuals [3]. The most common HIV-related lymphomas are diffuse large B-cell lymphoma (DLBCL), which includes primary CNS lymphoma (PCNSL), and Burkitt lymphoma (BL). Primary effusion lymphoma (PEL), and plasmablastic lymphoma (PBL) are less frequent, although they occur with preference in HIV-positive patients. Hodgkin lymphoma (HL) is a non-AIDS defining neoplasm, but PLWH have between a 5- and 26-fold higher risk of developing it than the general population. Unlike the dramatic decrease observed in the incidence of NHL among PLWH with the introduction of cART, the risk of HL initially increased but eventually has remained stable or decreased [2,4,5].

Classical HL (cHL) is the type that has been linked to PLWH. The most common histologic subtype is mixed cellularity followed by nodular sclerosis and lymphocyte-depleted [6,7,8,9]. Unlike cHL affecting the general population, around 90% of cases of PLWH-related are associated with Epstein–Barr virus (EBV) infection of tumor cells, which are Hodgkin Reed–Sternberg cells (HRS) [10]. The etiopathogenesis of HIV-related cHL is not yet fully understood. However, there is evidence indicating a crucial role for EBV infection of pre-apoptotic B cells, together with a cooperation with HIV, for triggering the lymphomagenic process [11,12]. Interactions between lymphoma cells and the microenvironment will eventually contribute to maintaining their proliferation as well as their escaping from the immune responses [11,13].

Although presenting with more aggressive characteristics, PLWH with cHL have similar response rates and survival to HIV-negative patients when they are treated with the same standard therapies [9,14]. Early and effective cART during chemotherapy has been shown to increase survival rates. Hence, initiation or maintenance of cART is highly recommended for PLWH with any type of lymphoma, including cHL [15,16,17]. As a consequence, it is currently very important to take into account the potential drug–drug interactions between antiretrovirals and drugs administered for the treatment of cHL.

In this article the authors review and update the epidemiological, clinical, and biological aspects of cHL presenting in PLWH with special emphasis on the improvement of prognosis and the factors that have contributed to it.

## 2. Epidemiology

The relative risk of HL in PLWH is 5- to 26-fold higher than in the general population with an estimated incidence of around 50 cases per 100,000 persons per year [4,18]. The subtype characteristically linked to HIV infection is cHL. Some studies show that PLWH at cHL diagnosis are older than those of the general population, such as one from the French ANRS-CO16 Lymphovir cohort (median of 44 vs. 29 years) [19] and other from the UK (median of 41 vs. 31 years) [14]. With the advent of cART, an increase in the incidence of HL was observed within the first few years. However, after the increment observed in the first decade, the incidence of HL eventually seems to have remained stable over the last few years [4,20,21]. In a collaborative work, including 33 observational cohort studies of adult and pediatric HIV-infected patients in 30 European countries, PLWH who develop HL had lower CD4 counts than controls (PLWH without lymphoma) [18]. However, at cHL diagnosis patients usually have a moderate decrease in CD4+ lymphocytes (between 150 and 260 cells/µL [22,23]. It has been speculated that this fact could be explained because of a certain number of CD4-positive lymphocytes are needed to facilitate the micro-environment development and the proliferation of HRS cells [2,24]. In turn, HRS cells produce many cytokines and chemokines, resulting in an influx of activated CD4 cells, histiocytes, and other cells. On the other hand, very low CD4 counts would lead to an impairment of these mechanisms and, hence, to a worse condition for the development of HL in severely immunosuppressed PLWH [23,25,26]. This hypothesis would explain the observation that the increase in the incidence of HL in the cART era has been observed mainly in those HIV-infected individuals with moderate immune suppression. On the other hand, the most immune suppressed individuals would be at lower risk of developing HL, but higher than those with CD4 counts above 0.5 × 10^9^/L who have a similar risk than the general population [23]. Of note, some studies reported that HIV-infected individuals are at higher risk of developing HL in the first 6 months after initiation of cART [27,28].

## 3. Etiopathogenesis

In cHL, the malignant HRS are scarce among an extensive and complex microenvironment. They are B cells because they carry immunoglobulin (Ig) heavy- and light-chain V gene rearrangements [29]. Their specific origin appears to be, in the majority of cases, pre-apoptotic germinal center (GC) B cells because destructive somatic mutations in the rearranged immunoglobulin (Ig) genes have been observed, leading to the loss of the capacity to express a B-cell receptor (BCR) [24]. The sequence of events during malignant transformation of pre-apoptotic GC B cells toward HRS cells is poorly understood, but escape from programmed cell death seems to be an early and essential event [30]. Nearly all cases of cHL with destructive Ig gene mutations eliminating BCR expression (e.g., nonsense mutations) are EBV-positive, suggesting that EBV-encoded genes have a particular function to prevent apoptosis of HRS-cell precursors that acquire these crippling mutations [31].

Virtually all cases of cHL in PLWH are EBV associated and show a type II latency pattern. They express viral proteins such as EBV nuclear antigen-1 (EBNA1), latent membrane protein 1 (LMP1), and LMP2, as well as EBERs and BARTs RNAs [32,33,34,35,36]. There is some evidence indicating a pathogenic role for EBV in the early stages of lymphomagenesis in EBV-positive cHL cases [11]. The protein EBNA1 is mandatory for the replication of the viral genome [24]. The expression of LMP1 and LMP2A (one of the two proteins encoded by *LMP2*), seems to play a crucial role in the development of EBV-related cHL [37,38]. LMP1 promotes B-cell activation and proliferation by activating NF-κB, mitogen-activated protein kinase (MAPK), phosphatidylinositol 3-kinase (PI3-K), IRF7, and STAT pathways [39]. This function is mainly produced because LMP1 mimics the CD40 receptor [34,40,41]. Interestingly, HIV virions from CD4+ cells harbor a CD40 ligand (CD40L) that might complement the effects induced by LMP1 [42,43,44]. On the other hand, LMP2A prevents apoptosis via mimicking B-cell receptor (BCR) signaling [45,46]. In addition, EBV induces the overexpression of PD-L1 in a subset of cHL cases, leading to an immune escape response and contributing altogether to EBV-infected HRS proliferation and tumor progression [47].

The implication of EBV seems to be a higher influence on the microenvironment of cHL, as EBV-positive cHL tissues are enriched in genes characteristic of T-cell and antiviral responses. The cellular microenvironment of EBV-positive cHL cells is largely composed of immune cells that are probably attempting to eliminate EBV-positive HRS cells, together with inflammatory cells that contribute to the growth of the neoplastic component [11,13]. Cytotoxic T lymphocytes have been isolated from cHL patients and have been shown to specifically kill LMP1 and LMP2 expressing targets ex vivo [48,49]. Moreover, high numbers of CD4+ CD25+ regulatory T cells (Tregs) have been detected in the peripheral blood and tumor tissues of cHL patients [48,49,50,51,52]. The proteins EBNA1 and LMP1 have been demonstrated to play a role in attracting Tregs to the cHL tissue [53,54]. Additionally, high numbers of CD8+ and natural killer cells have been identified in tissues of cHL cases [45]. Therefore, it seems that in EBV-positive cHL, activated CD8+ T cells, probably specific for viral epitopes, and Treg cells coexist in the microenvironment [11].

Compared to EBV-negative cases, EBV-related cHL have higher infiltration by macrophages, mainly of type M1, which promote Th1 responses and kill tumor cells [55,56,57,58]. There is some evidence indicating that this macrophage infiltrating pattern is also predominant in cHL in the HIV setting [59,60]. A differential characteristic of the HIV-related cHL microenvironment is the paucity of CD4+ cells in the infiltrate surrounding HRS cells [61,62]. This is likely due to the reduced CD4+ lymphocyte count present in PLWH at cHL diagnosis. Moreover, a significant reduction in CD56+ cells (functional NK cells), CD57+ cells (terminally differentiated T lymphocytes and mature NK), CD123+ plasmocytoid dendritic cells, and B cells, have been observed [62]. These findings show the differences between the microenvironment of cHL in PLWH and that of the general population and could contribute to the increased incidence of cHL among HIV-infected people [62]. On the other hand, the absolute number of CD8+ T lymphocytes is preserved in these cases, although a decrease in infiltrating GrB+ cells (activated cytotoxic cells) and an increase in infiltrating TIA+ T cells (mainly nonactivated cytotoxic cells) are observed [59,61]. It has been speculated that these differences in the cellular components of the microenvironment could be due to the specific cytokine/chemokine profile of HIV-related cHL [11].

A cooperation between HIV and EBV has been speculated to take part in lymphomagenesis, through interactions mediated by cellular dysregulation/immunodeficiency and/or chronic antigenic stimulation/inflammation [12]. Regarding this, some HIV-encoded proteins and the virus itself promote B-cell proliferation and activation by chronic antigenic stimulation [63,64,65]. This would lead to an oligoclonal dysregulated B-cell expansion that would be at risk of acquiring genetic alterations, finally leading to lymphoma development [11]. The hyperactivated B cells, induced either directly or indirectly by HIV stimuli, may express activation-induced cytidine deaminase (AID), a DNA editing enzyme that mediates immunoglobulin class switch recombination, somatic hypermutation, and the development of chromosomal translocations [66,67].

In summary, the lymphomagenesis of cHL in PLWH seems to be the result of interactions between pre-apoptotic B cells and the microenvironment, and the cooperation of both viruses, EBV and HIV, along with the presence of inherent genetic abnormalities. These mechanisms might trigger lymphomagenesis by activating cell signaling pathways. The interactions between HRS cells and the microenvironment will eventually develop and maintain malignant cell growth.

## 4. Pathological and Clinical Characteristics

The WHO classification of tumors of hematopoietic and lymphoid tissues considers two types of HL with different pathological characteristics; nodular lymphocyte predominant HL and classical HL (cHL), which is the type associated to HIV infection [68]. From the 4 histological cHL subtypes, the most frequent among PLWH is mixed cellularity followed by nodular sclerosis [6,9,69].

**Figure 1:**
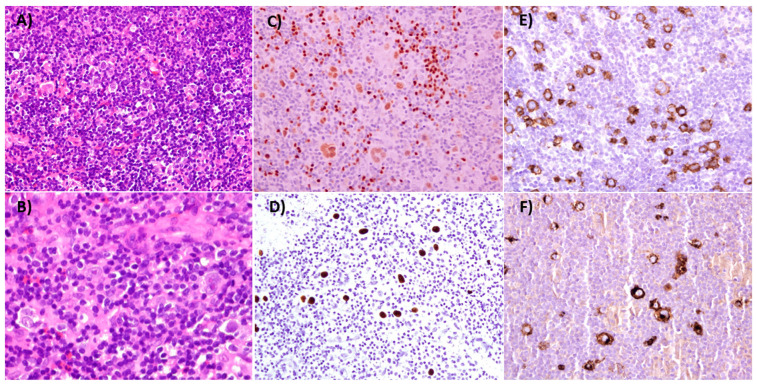
Classical Hodgkin Lymphoma, Mixed Cellularity. The lymph node architecture is effaced by a mixed population of lymphocytes, plasma cells, eosinophils, histiocytes and Reed–Sternberg (RS) cells ((**A**,**B**), Hematoxilin & eosin, 100× and 400×). RS cells are weakly positive for PAX5 ((**C**), 200×), and Epstein–Barr encoded RNA (EBERs) can be detected ((**D**), 200×). CD30 and CD15 are strongly positive in RS cells ((**E**,**F**) respectively, 200×).

Pathology findings are similar in HIV-positive and HIV-negative patients. In both settings, HRS are characteristically observed on a heterogeneous background of lymphocytes, eosinophils, neutrophils, macrophages, and plasma cells. Neoplastic cells show the usual HRS phenotype (PAX5+, CD30+, CD15+), rarely express CD20 and usually are CD45 negative. The frequency of mixed cellularity subtype increases along with the decrease of CD4+ lymphocytes [23]. Coinfection with EBV occurs in 90–100% of cases compared to 30–40% in HIV-negative patients [11,24,68]. The HRS cells express EBNA1, LMP1 and are EBER positive. Figure 1 shows a typical case of mixed cellularity in an HIV-positive case.

A characteristic pathological finding in HIV-related cHL is a higher amount of HRS cells compared with cHL in HIV-negative patients [11]. The presence of large confluent areas of necrosis underlying the presence of a proinflammatory activity has been also described, with a “sarcomatoid pattern”, attributed to the increased quantity of CD163 spindle shaped macrophages [59]. The most typical feature of HIV-related cHL is likely the scarce number of CD4+ T cells present in the microenvironment and an inverted CD4/CD8 T-cell ratio resulting in a predominance of CD8+ T lymphocytes in the background [59,61,62].

Regarding the clinical features, the proportion of males is higher than in HIV-negative subjects and some studies have shown that the age at diagnosis is higher in PLWH [14,21]. Among PLWH, cHL often presents with unfavorable features at diagnosis, such as poorer performance status, advanced-stage, extranodal disease, and bone marrow involvement [9,14,19]. The presence of B symptoms is also more frequent than in the general population [14] and exclusive extranodal presentation has been reported in some sites such as bone marrow and liver [70,71]. In the cART era the median CD4 count at HL diagnosis is between 120 and 385 × 10^9^/L [6,9,14] (Table 1).

## 5. Treatment and Prognosis

Before starting the treatment, a staging procedure, including the same tests as in HIV-negative patients, should be performed. A basal PET-CT scan is mandatory in all cases, but bone marrow biopsy can be avoided in most cases due to the reliability of PET-CT in diagnosing infiltration by cHL in this site.

With the introduction of cART, the prognosis of PLWH and cHL has been steadily improving until patients have reached almost the same outcomes as cHL in the general population when applying the same treatments [6,9,14,74]. Some studies, performed in the cART era, have shown that response rates and survival of cHL in PLWH are similar to those in HIV-negative patients, although HIV-patients presented more aggressive characteristics [9,14,19,75,76]. In our own experience, the results are good even in patients with low CD4+ lymphocyte counts. In the study by Xicoy et al., we did not find worse outcomes in patients with CD4+ lymphocyte <200/µL treated with ABVD [6].

For this reason, the recommendations for the treatment of cHL in PLWH should not differ from those in the general population. Standard regimens such as ABVD (doxorubicin, bleomycin, vinblastine and dacarbazine), BEACOPP (bleomycin, etoposide, doxorubicine, cyclophosphamide, vincristine, procarbazine, prednisone) baseline and Stanford V have been demonstrated to be highly effective in HIV-infected patients [6,9,14,72,73,74]. In a retrospective study comparing PLWH and HIV-negative individuals treated with ABVD, the complete response (CR) rates were 74% and 79%, respectively, and five-year overall survival (OS) was 81% and 88% for HIV-positive and HIV-negative patients, respectively [14]. Results from the French cohort reported again no differences between HIV-negative and HIV-positive patients [19] In a similar study, patients with advanced cHL treated with ABVD, had similar CR rates, (89% in HIV-positive vs. 91% in HIV-negative) and survival. In all these studies, HIV-positive patients received cART concomitantly with ABVD. Moreover, Yotsumoto and colleagues, compared only EBV-positive HL cases, most of them treated with ABVD (with or without radiotherapy) and did not find significant differences in the CR rate, OS, and progression-free survival (PFS) between EBV+ HIV-positive and EBV+ HIV negative instances. However, in this study, whether HIV-positive patients received cART along with chemotherapy was not reported [76].

As in HIV-negative patients, the treatment can be tailored by taking into account the risk factors and stratifying the patients, aiming for less toxicity and high efficacy. In this sense, Hentrich et al. reported a study administering different treatment to patients with early-stage with favorable risk HL (2 cycles of ABVD followed by 20 Gy of involved-field radiotherapy) than to those with early-stage with unfavorable risk (4 cycles of BEACOPP baseline followed by 30 Gy of involved-field radiotherapy) [74]. In this study, advanced stage patients received 6–8 cycles of BEACOPP baseline. The results of these approaches showed similar outcomes to those reported in the general population. However some patients with advanced disease died because of neutropenic infections related to treatment toxicity, meaning that this regimen should be given with caution in PLWH [74]. On the other hand, due to the lack of prospective studies, there is scarce reliable information on toxicity of ABVD in PLWH. However, based on the available information, this regimen seems to be safe with acceptable toxicity in the HIV-setting [6,14]. Table 1 summarizes the results of front-line treatment of cHL in PLWH of the main studies performed in the cART era.

Interim PET-CT after two or three cycles can be used to decide if less chemotherapy can be given according to the to the metabolic response. A retrospective study by Lawal et al. showed the usefulness of fluorine-18-fluorodeoxyglucose PET (FDG-PET) performed at diagnosis to stratify PLWH and cHL, without differences in metabolic parameters between HIV-positive and HIV-negative patients [77]. A study by Okosun et al. demonstrated the utility of an interim FDC-PET after two or three cycles to predict outcomes in PLWH with advanced stage cHL treated with BVD [78]. They reported 100% 2-year PFS probability in patients with negative interim PET-CT. Other studies have shown the feasibility of stage-adapted approach treatments in the HIV-setting based on an interim PET-CT. Danilov et al. reported the usefulness of an interim PET-CT after two cycles of ABVD to guide further treatment in HIV-infected individuals with advanced HL. In this study 10 patients with negative interim PET-CT were scheduled to receive four additional cycles of ABVD. Nine of them completed the six cycles and only one patient discontinued it due to disease progression [79].

Brentuximab vedotin (BV) is an anti-CD30 antibody-drug conjugate potently active in Hodgkin lymphoma, approved by the Food and Drug Administration and the European Medicines Agency for frontline treatment of HL in combination with doxorubicin, vinblastine, and dacarbazine (AVD-BV). However, as usual, trials excluded HIV-infected patients and the usefulness of BV in PLWH with cHL is still under investigation. A phase I trial demonstrated the combination AVD-BV was well tolerated with 100% CR, in the absence of strong CYP3A4 inhibitors as part of cART, and a phase II trial is ongoing [80].

Relapses in HIV-infected patients with cHL can be treated with the same strategies as HIV-negative patients including autologous stem cell transplantation. Several studies have reported similar outcomes in HIV-infected patients and the general population when treated with salvage therapy followed by autologous stem cell transplantation [81,82,83]. Moreover, two patients with cHL have been reported in a prospective clinical trial of allogeneic bone marrow transplantation for patients with HIV and hematological malignancies [84].

The new immunomodulatory treatments, such as checkpoint inhibition with anti-PDL1 drugs, have been used in some patients and are currently under investigation in a clinical trial combining Nivolumab, an anti-PD-1 blocking antibody, and Ipilimumab, a monoclonal antibody against CTLA-4 (NCT02408861) [85,86].

### Additional Measures and Supportive Care

In addition to specific lymphoma treatment, there are other issues to take into account in the management of HIV-related lymphomas. Antimicrobial systematic prophylaxis is a matter of controversy. Some groups are in favor of using fluoroquinolones, but this practice is not generally recommended, because of the concern of generating bacterial resistances to antibiotics and side effects [15,87]. However, primary infectious prophylaxis using colony-stimulating factors such as G-CSF given after every cycle of chemotherapy, is highly recommended [15,17,88]. Prophylaxis against *Pneumocystis jirovecii* should be given to all PLWH who receive chemotherapy or radiotherapy as these treatments have been demonstrated to decrease CD4+ lymphocyte counts [89,90,91]. The most recommended is cotrimoxazole, which may have the additional benefits of prevention from bacterial infections and toxoplasmosis. *Mycobacterium avium* complex should also be prevented in patients with CD4+ lymphocytes lower than 50/µL, using oral azithromycin [88,92].

## 6. Management of cART in Patients with Classical Hodgkin Lymphoma

### 6.1. Initiation/Maintenance of cART

Whether combining cART with chemotherapy outweighs potential risk of increased toxicity has remained controversial. The risk of overlapping toxicities and the potential for difficult-to-manage drug–drug interactions have been reasons to justify postponement or interruption of cART during chemotherapy by some authors [92,93]. However, effective cART during chemotherapy has been shown to improve survival in PLWH with lymphoma [94,95,96,97,98,99]. Gopal and colleagues reported a 35% increase in mortality 5 years after lymphoma diagnosis for each log10 increase in plasma HIV RNA load within the 6 months after lymphoma diagnosis [97]. In addition, interruption of cART has been associated with higher risk of death, AIDS, and serious non-AIDS morbidity [100]. Consequently, initiation or maintenance of cART is currently recommended for PLWH with cancer, including cHL [101]. One possible exception to this statement would be the case of patients with a very poor prognosis. In such patients it may be reasonable to forego cART since they are unlikely to have either HIV-related symptoms or a survival benefit from the addition of cART.

### 6.2. Drug Interactions between cART and Chemotherapy

Currently approved antiretroviral drugs include nucleos(t)ide and non-nucleoside reverse-transcriptase inhibitors (NRTIs and NNRTIs, respectively), protease inhibitors (PIs), integrase strand transfer inhibitors (INSTIs), and entry inhibitors [102]. Management of PLWH with cHL remains challenging due to potential drug–drug interactions among antineoplastics, co-medications and antiretroviral drugs.

Despite the lack of controlled studies, clinically significant interactions between chemotherapy regimens and cART have been reported. The risk for interactions is highest with antiretroviral regimens that include ritonavir or cobicistat (commonly known as “boosters”). The use of boosters aims to increase concentrations in plasma of other antiretrovirals including PIs or the INSTI elvitegravir to attain therapeutic concentrations over 24 h. However, ritonavir and cobicistat are potent inhibitors of cytochrome P450 enzymes and drug transporters which are involved in the disposition of numerous drugs, leading to marked increases in drug exposure [103,104]. Specifically in PWLH with lymphoma, the use of boosters have been associated with a higher probability of dose-reduction and treatment delay as well as with worse OS [105,106]. Specifically, the use of ritonavir was shown to raise the risk of both hematologic and nonhematologic adverse events in PLWH treated with cyclophosphamide, doxorubicin and etoposide [107,108]. Similarly, Leveque et al. described increased autonomic neurotoxicity in one patient receiving lopinavir/ritonavir and vincristine [109]. All of these limitations together with current availability of other cART options with similar efficacy and better tolerability mean that unboosted regimens should be considered for PLWH undergoing chemotherapy for lymphoma.

In patients with lymphoma unboosted INSTIs may be particularly recommended due to their favorable interaction profile with antineoplastic drugs. Raltegravir, dolutegravir or bictegravir do not exert inducer or inhibitor effects on P450 enzymes or drug transporters, minimizing their potential for drug interactions [110,111,112]. Conversely, elvitegravir needs to be coadministered with cobicistat. For this reason, the use of elvitegravir-based cART in PLWH receiving chemotherapy shares most of the limitations of boosted PIs, and its use in this setting should be discouraged.

On the contrary to ritonavir or cobicistat, some NNRTIs (i.e., nevirapine, efavirenz, etravirine) are moderate to potent inducers of cytochrome P450 enzymes, and could potentially reduce exposure, and thus efficacy, of certain chemotherapy drugs [113]. Rilpivirine and doravirine are second-generation NNRTIs that do not induce the P450 system limiting their potential for interactions with chemotherapy [114,115].

Nucleoside analogues reverse-transcriptase inhibitors are still considered the backbone of cART [102]. Although no pharmacokinetic interactions between NRTIs and chemotherapy are expected, their concomitant use with chemotherapy may be limited by pharmacodynamic interactions with overlapping toxicity. Tenofovir may be associated with renal toxicity [116]. Thus, if the patient is receiving tenofovir disoproxil fumarate with other potentially nephrotoxic drugs (i.e., methotrexate, cisplatin, etc.). the use of tenofovir alafenamide may be preferred. Similarly, zidovudine may cause anemia, myelosuppression, fatigue and nausea; and patients treated with didanosine or stavudine may develop peripheral neuropathy, which can be worsened by chemotherapy [102].

Beside causing drug interactions, antiretroviral drugs may also be victims of interactions caused by co-medications commonly used in patients with lymphoma. For example, omeprazole and other proton pump inhibitors may reduce oral bioavailability of rilpivirine, and coadministration may result in the loss of the therapeutic effect of rilpivirine [114]. Antiacids or multivitamins containing divalent cations may decrease oral absorption of INTIs if they are taken at the same time, and dose staggering should be recommended [117,118].

### 6.3. Clinical Approach to Management of Patients on cART and Hodgkin Lymphoma

Since standard dosing algorithms do not exist for managing interactions between cART and chemotherapy, increased monitoring for safety and efficacy is strongly recommended in PLWH undergoing chemotherapy for cHL. In addition, the risk of specific drug–drug interactions between antiretroviral and antineoplastic or supportive drugs should be addressed. In this regard, we recommend consulting specific web pages on this topic, such as www.hiv-druginteractions.org (accessed on 30 May 2021) [119] or www.hivclinic.ca/main/drugs_interact.html (accessed on 30 May 2021) [120] (Table 2).

A stable antiretroviral regimen can be modified before chemotherapy to avoid drug–drug interactions, reduce toxicity, and improve adherence and tolerability. As abovementioned, discontinuation of ritonavir or cobicistat-containing regimens in favor of unboosted INSTIs should be encouraged. However, the discontinuation of a single drug in the antiretroviral regimen thought to interact with chemotherapy must be avoided, as this may decrease the efficacy of cART and promote the development of viral resistance to the other antiretrovirals that are to be continued. Changes in cART should be made in consultation with an HIV specialist, since knowledge of the patient’s complete treatment history, including resistance data is crucial when designing alternative cART options. Therefore, interdisciplinary collaboration for the optimal treatment of the oncologic process and HIV infection is mandatory [22,121,122], and may result in better outcomes for PLWH with HL, including better PFS rates (personal communication, unpublished).

## 7. Conclusions

The widespread use of cART initially produced an increase in the incidence of cHL in PLWH. The etiopathogenesis of this lymphoma in the HIV-setting has some differential characteristics due to HIV and EBV cooperation and the different composition of the microenvironment compared to non-HIV patients. Although more aggressive clinical features are still present in cHL affected PLWH, the prognosis has improved and is currently similar to that of HIV-negative patients. The therapeutic approach for HIV-related cHL should not differ from that for the general population. The standard strategies used in the general population to treat cHL have been shown to be equally effective among PLWH. Patients with HIV-related cHL should be placed or maintained on cART during treatment. However, the concomitant administration of chemotherapy with cART may be challenging due to drug–drug interactions and overlapping toxicity. Thus, interdisciplinary collaboration between hemato-oncologists and HIV specialists is crucial for the optimal treatment of both lymphoma and HIV infection while minimizing the risk of adverse outcomes for the patient.

## Figures and Tables

**Table 1:** Results of front-line treatment of the main studies performed on cHL in PLWH since the introduction of cART.

Author	Chemotherapy Regimen	N	Median Age * (Range)	Stage	CD4+ Count/µL * Median (Range)	CR (%)	Survival (%)	Overall Survival (%)
Spina et al. [72]	Stanford V	59	38 (28–64)	I–IV	238 (32–1038)	81	68 (3-year DFS)	51 (3-year)
Hartmann et al. [73]	BEACOPP	12	33 (22–49)	III–IV	205 (110–1020)	100	70 (5-year DFS)	70 (5-year)
Xicoy et al. [6]	ABVD	51	37 (24–61)	II–IV	129 (5–1209)	87	95 (5-year EFS)	76 (5-year)
Montoto et al. [14]	ABVD	93	41 (26–73)	I–IV	NA	74	59 (5-year EFS)	81 (5-year)
Hentrich et al. [74] ^1^	BEACOPP baseline or ABVD ^2^ Stage-adapted	71/108	44 (27–70) ^3^	III–IV	240 (7–967) ^3^	86 ^1^	87.5 (2-year PFS) ^1^	87 (2-year) ^1^
Castillo et al. [75]	ABVD	229	NA	III–IV	NA	83	69 (5-year PFS)	78 (5-year)
Besson et al. [19]	ABVD (96%)	68	44 (38–48)	I–IV	387 (151–540)	NA	89 (2-year PFS	94 (2-year)
Sorigué et al. [9]	ABVD	21	40 (18–56)	III–IV	NA	89	70 (10-year DFS)	73 (10-year)

* Age and CD4+ count at HL diagnosis. ^1^ Treatment results refer only to advanced stage cases (III-IV, N = 71). ^2^ ABVD was given in advanced stage if CD4 < 50/µL. ^3^ results refer to the whole series (N = 108); ABVD: adriamycin-bleomycin-vinblastine-dacarbazine BEACOPP: bleomycin-etoposide-doxorubicin (adryamicine)-cyclophosphamide-vincristine (oncovin)-procarbacine-prednisone; CR: complete response; DFS: disease-free survival (calculated for patients with CR from the first CR recorded until relapse or until the last known date on which the patient was disease-free); EFS: event-free survival (defined for all patients as time from diagnosis to failure of treatment, including not achieving CR/CR uncertain or relapse after CR/CR uncertain, or death from any cause); NA: not available; PFS: progression-free survival (defined as the time between the date of diagnosis and the date of progression, death, or last follow-up.

**Table 2:** Main drug–drug interactions (DDI) between drugs for the treatment of Hodgkin lymphoma and antiretroviral agents (www.hiv-druginteractions.org; www.hivclinic.ca/main/drugs_interact.html accessed on 30 May 2021) *.

	DRV/r DRV/c	ATV/r ATV/c	LPV/r	NVP	EFV	ETR	RPV	DOR	RAL	EVG/c	DTG	BIC
**Cyclophosphamide (CYC)**	Monitor CYC toxicity	Monitor CYC toxicity	Monitor CYC toxicity	Monitor CYC efficacy/toxicity	Monitor CYC efficacy/toxicity	Monitor CYC efficacy/toxicity	No DDI expected	No DDI expected	No DDI expected	Monitor CYC toxicity	No DDI expected	No DDI expected
**Doxorubicin (DOX)**	No DDI expected	Monitor ECG **	Monitor ECG **	No DDI expected	No DDI expected	No DDI expected	Monitor ECG **	No DDI expected	No DDI expected	No DDI expected	No DDI expected	No DDI expected
**Vincristine/Vinblastine (VIN)**	Increased VIN toxicity	Increased VIN toxicity	Increased VIN toxicity	Monitor VIN efficacy	Monitor VIN efficacy	Monitor VIN efficacy	No DDI expected	No DDI expected	No DDI expected	Increased VIN toxicity	No DDI expected	No DDI expected
**Prednisone (PRE)**	Monitor PRE toxicity	Monitor PRE toxicity	Monitor PRE toxicity	Monitor PRE efficacy	Monitor PRE efficacy	Monitor PRE efficacy	No DDI expected	No DDI expected	No DDI expected	Monitor PRE toxicity	No DDI expected	No DDI expected
**Etoposide (ETO)**	Monitor ETO toxicity	Monitor ETO toxicity	Monitor ETO toxicity	Monitor ETO efficacy	Monitor ETO efficacy	Monitor ETO efficacy	No DDI expected	No DDI expected	No DDI expected	Monitor ETO toxicity	No DDI expected	No DDI expected
**Bleomycin (BLE)**	No DDI expected	No DDI expected	No DDI expected	No DDI expected	No DDI expected	No DDI expected	No DDI expected	No DDI expected	No DDI expected	No DDI expected	No DDI expected	No DDI expected
**Brentuximab (BRE)**	Monitor BRE toxicity	Monitor BRE toxicity	Monitor BRE toxicity	No DDI expected	No DDI expected	No DDI expected	No DDI expected	No DDI expected	No DDI expected	Monitor BRE toxicity	No DDI expected	No DDI expected
**Dacarbazine (DAC)**	Monitor DAC toxicity	Monitor DAC toxicity	Monitor DAC toxicity	No DDI expected	No DDI expected	No DDI expected	No DDI expected	No DDI expected	No DDI expected	No DDI expected	No DDI expected	No DDI expected
**Nivolumab (NIV)**	No DDI expected	No DDI expected	No DDI expected	No DDI expected	No DDI expected	No DDI expected	No DDI expected	No DDI expected	No DDI expected	No DDI expected	No DDI expected	No DDI expected
**Pembrolizumab (PEM)**	No DDI expected	No DDI expected	No DDI expected	No DDI expected	No DDI expected	No DDI expected	No DDI expected	No DDI expected	No DDI expected	No DDI expected	No DDI expected	No DDI expected
**Procarbazine (PRO)**	Monitor PRO efficacy	Monitor PRO efficacy	Monitor PRO efficacy	Monitor PRO efficacy	Monitor PRO efficacy	No DDI expected	No DDI expected	No DDI expected	No DDI expected	No DDI expected	No DDI expected	No DDI expected
**Rituximab (RIT)**	No DDI expected	No DDI expected	No DDI expected	No DDI expected	No DDI expected	No DDI expected	No DDI expected	No DDI expected	No DDI expected	No DDI expected	No DDI expected	No DDI expected

DRV/r: darunavir/ritonavir; DRV/c: darunavir/cobicistat; ATV/r: atazanavir/ritonavir; ATV/c: atazanavir/cobicistat;LPV/r: lopinavir/ritonavir; NVP: nevirapine; EFV: Efavirenz; ETR: etravirine; RPV: rilpivirine; DOR: doravirine; RAL: raltegravir; EVG/c: elvitegravir/cobicistat; DTG: dolutegravir; BIC: bictegravir. * Coadministration of most of these drugs has not been studied. Potential DDI are based on theoretical data. ** Monitor QT interval in the ECG with lopinavir/ritonavir, atazanavir and rilpivirine.

## References

[1] Shiels M.S., Pfeiffer R.M., Hall H.I., Li J., Goedert J.J., Morton L.M., Hartge P., Engels E.A. (2011). Proportions of Kaposi Sarcoma, Selected Non-Hodgkin Lymphomas, and Cervical Cancer in the United States Occurring in Persons with AIDS, 1980–2007. JAMA.

[2] Kimani S.M., Painschab M.S., Horner M.-J., Muchengeti M., Fedoriw Y., Shiels M.S., Gopal S. (2020). Epidemiology of Haematological Malignancies in People Living with HIV. Lancet HIV.

[3] Horner M.-J., Shiels M.S., Pfeiffer R.M., Engels E.A. (2021). Deaths Attributable to Cancer in the US Human Immunodeficiency Virus Population During 2001-2015. Clin. Infect. Dis..

[4] Hernández-Ramírez R.U., Shiels M.S., Dubrow R., Engels E.A. (2017). Cancer Risk in HIV-Infected People in the USA from 1996 to 2012: A Population-Based, Registry-Linkage Study. Lancet HIV.

[5] Robbins H.A., Shiels M.S., Pfeiffer R.M., Engels E.A. (2014). Epidemiologic Contributions to Recent Cancer Trends among HIV-Infected People in the United States. AIDS.

[6] Xicoy B., Ribera J.-M., Miralles P., Berenguer J., Rubio R., Mahillo B., Valencia M.-E., Abella E., López-Guillermo A., Sureda A. (2007). Results of Treatment with Doxorubicin, Bleomycin, Vinblastine and Dacarbazine and Highly Active Antiretroviral Therapy in Advanced Stage, Human Immunodeficiency Virus-Related Hodgkin’s Lymphoma. Haematologica.

[7] Díez-Martín J.L., Balsalobre P., Re A., Michieli M., Ribera J.M., Canals C., Conde E., Rosselet A., Gabriel I., Varela R. (2009). Comparable Survival between HIV+ and HIV- Non-Hodgkin and Hodgkin Lymphoma Patients Undergoing Autologous Peripheral Blood Stem Cell Transplantation. Blood.

[8] Ruiz M., Parsons C., Cole J. (2012). Characterization of HIV-Associated Hodgkin’s Lymphoma in HIV-Infected Patients: A Single-Center Experience. J. Int. Assoc. Physicians AIDS Care.

[9] Sorigué M., García O., Tapia G., Baptista M.-J., Moreno M., Mate J.-L., Sancho J.M., Feliu E., Ribera J.-M., Navarro J.-T. (2017). HIV-Infection Has No Prognostic Impact on Advanced-Stage Hodgkin Lymphoma. AIDS.

[10] Mani H., Jaffe E.S. (2009). Hodgkin Lymphoma: An Update on Its Biology with New Insights into Classification. Clin. Lymphoma Myeloma.

[11] Carbone A., Gloghini A., Caruso A., Paoli P.D., Dolcetti R. (2017). The Impact of EBV and HIV Infection on the Microenvironmental Niche Underlying Hodgkin Lymphoma Pathogenesis. Int. J. Cancer.

[12] De Paoli P., Carbone A. (2015). Microenvironmental Abnormalities Induced by Viral Cooperation: Impact on Lymphomagenesis. Semin. Cancer Biol..

[13] Chetaille B., Bertucci F., Finetti P., Esterni B., Stamatoullas A., Picquenot J.M., Copin M.C., Morschhauser F., Casasnovas O., Petrella T. (2009). Molecular Profiling of Classical Hodgkin Lymphoma Tissues Uncovers Variations in the Tumor Microenvironment and Correlations with EBV Infection and Outcome. Blood.

[14] Montoto S., Shaw K., Okosun J., Gandhi S., Fields P., Wilson A., Shanyinde M., Cwynarski K., Marcus R., de Vos J. (2012). HIV Status Does Not Influence Outcome in Patients with Classical Hodgkin Lymphoma Treated with Chemotherapy Using Doxorubicin, Bleomycin, Vinblastine, and Dacarbazine in the Highly Active Antiretroviral Therapy Era. J. Clin. Oncol..

[15] Bower M., Palfreeman A., Alfa-Wali M., Bunker C., Burns F., Churchill D., Collins S., Cwynarski K., Edwards S., Fields P. (2014). British HIV Association Guidelines for HIV-Associated Malignancies 2014. HIV Med..

[16] Hentrich M., Hoffmann C., Mosthaf F., Müller M., Siehl J., Wyen C., Hensel M., German Study Group of Physicians in Private Practice Treating HIV-Infected Patients (DAGNÄ), German AIDS Society (DAIG) (2014). Therapy of HIV-Associated Lymphoma-Recommendations of the Oncology Working Group of the German Study Group of Physicians in Private Practice Treating HIV-Infected Patients (DAGNÄ), in Cooperation with the German AIDS Society (DAIG). Ann. Hematol..

[17] Miralles P., Navarro J.T., Berenguer J., Gómez Codina J., Kwon M., Serrano D., Díez-Martín J.L., Villà S., Rubio R., Menárguez J. (2018). GESIDA/PETHEMA Recommendations on the Diagnosis and Treatment of Lymphomas in Patients Infected by the Human Immunodeficiency Virus. Med. Clin..

[18] Bohlius J., Schmidlin K., Boué F., Fätkenheuer G., May M., Caro-Murillo A.M., Mocroft A., Bonnet F., Clifford G., Paparizos V. (2011). HIV-1–Related Hodgkin Lymphoma in the Era of Combination Antiretroviral Therapy: Incidence and Evolution of CD4+ T-Cell Lymphocytes. Blood.

[19] Besson C., Lancar R., Prevot S., Brice P., Meyohas M.-C., Marchou B., Gabarre J., Bonnet F., Goujard C., Lambotte O. (2015). High Risk Features Contrast With Favorable Outcomes in HIV-Associated Hodgkin Lymphoma in the Modern CART Era, ANRS CO16 LYMPHOVIR Cohort. Clin. Infect. Dis..

[20] Calabresi A., Ferraresi A., Festa A., Scarcella C., Donato F., Vassallo F., Limina R., Castelli F., Quiros-Roldan E., Brescia HIV Cancer Study Group (2013). Incidence of AIDS-Defining Cancers and Virus-Related and Non-Virus-Related Non-AIDS-Defining Cancers among HIV-Infected Patients Compared with the General Population in a Large Health District of Northern Italy, 1999–2009. HIV Med..

[21] Hleyhel M., Hleyhel M., Bouvier A.M., Belot A., Tattevin P., Pacanowski J., Genet P., De Castro N., Berger J.-L., Dupont C. (2014). Risk of Non-AIDS-Defining Cancers among HIV-1-Infected Individuals in France between 1997 and 2009: Results from a French Cohort. AIDS.

[22] Re A., Cattaneo C., Rossi G. (2019). Hiv and Lymphoma: From Epidemiology to Clinical Management. Mediterr. J. Hematol. Infect. Dis..

[23] Biggar R.J., Jaffe E.S., Goedert J.J., Chaturvedi A., Pfeiffer R., Engels E.A. (2006). Hodgkin Lymphoma and Immunodeficiency in Persons with HIV/AIDS. Blood.

[24] Bräuninger A., Schmitz R., Bechtel D., Renné C., Hansmann M.-L., Küppers R. (2006). Molecular Biology of Hodgkin’s and Reed/Sternberg Cells in Hodgkin’s Lymphoma. Int. J. Cancer.

[25] van den Berg A., Visser L., Poppema S. (1999). High Expression of the CC Chemokine TARC in Reed-Sternberg Cells: A Possible Explanation for the Characteristic T-Cell Infiltrate in Hodgkin’s Lymphoma. Am. J. Pathol..

[26] Skinnider B.F., Mak T.W. (2002). The Role of Cytokines in Classical Hodgkin Lymphoma. Blood.

[27] Gotti D., Danesi M., Calabresi A., Ferraresi A., Albini L., Donato F., Castelli F., Scalzini A., Quiros-Roldan E., Brescia HIV Cancer Study Group (2013). Clinical Characteristics, Incidence, and Risk Factors of HIV-Related Hodgkin Lymphoma in the Era of Combination Antiretroviral Therapy. AIDS Patient Care STDs.

[28] Lanoy E., Rosenberg P.S., Fily F., Lascaux A.-S., Martinez V., Partisani M., Poizot-Martin I., Rouveix E., Engels E.A., Costagliola D. (2011). HIV-Associated Hodgkin Lymphoma during the First Months on Combination Antiretroviral Therapy. Blood.

[29] Kuppers R., Rajewsky K., Zhao M., Simons G., Laumann R., Fischer R., Hansmann M.L. (1994). Hodgkin Disease: Hodgkin and Reed-Sternberg Cells Picked from Histological Sections Show Clonal Immunoglobulin Gene Rearrangements and Appear to Be Derived from B Cells at Various Stages of Development. Proc. Natl. Acad. Sci. USA.

[30] Weniger M.A., Küppers R. (2021). Molecular Biology of Hodgkin Lymphoma. Leukemia.

[31] Braeuninger A., Küppers R., Strickler J.G., Wacker H.-H., Rajewsky K., Hansmann M.-L. (1997). Hodgkin and Reed–Sternberg Cells in Lymphocyte Predominant Hodgkin Disease Represent Clonal Populations of Germinal Center-Derived Tumor B Cells. Proc. Natl. Acad. Sci. USA.

[32] Audouin J., Diebold J., Pallesen G. (1992). Frequent Expression of Epstein-Barr Virus Latent Membrane Protein-1 in Tumour Cells of Hodgkin’s Disease in HIV-Positive Patients. J. Pathol..

[33] Carbone A., Gloghini A., Larocca L.M., Antinori A., Falini B., Tirelli U., Dalla-Favera R., Gaidano G. (1999). Human Immunodeficiency Virus–Associated Hodgkin’s Disease Derives From Post–Germinal Center B Cells. Blood.

[34] Linke-Serinsöz E., Fend F., Quintanilla-Martinez L. (2017). Human Immunodeficiency Virus (HIV) and Epstein-Barr Virus (EBV) Related Lymphomas, Pathology View Point. Semin. Diagn. Pathol..

[35] Deacon E.M., Pallesen G., Niedobitek G., Crocker J., Brooks L., Rickinson A.B., Young L.S. (1993). Epstein-Barr Virus and Hodgkin’s Disease: Transcriptional Analysis of Virus Latency in the Malignant Cells. J. Exp. Med..

[36] Niedobitek G., Kremmer E., Herbst H., Whitehead L., Dawson C.W., Niedobitek E., von Ostau C., Rooney N., Grässer F.A., Young L.S. (1997). Immunohistochemical Detection of the Epstein-Barr Virus-Encoded Latent Membrane Protein 2A in Hodgkin’s Disease and Infectious Mononucleosis. Blood.

[37] Young L.S., Murray P.G. (2003). Epstein-Barr Virus and Oncogenesis: From Latent Genes to Tumours. Oncogene.

[38] Kapatai G., Murray P. (2007). Contribution of the Epstein Barr Virus to the Molecular Pathogenesis of Hodgkin Lymphoma. J. Clin. Pathol..

[39] Kieser A., Sterz K.R. (2015). The Latent Membrane Protein 1 (LMP1). Curr. Top. Microbiol. Immunol..

[40] Gires O., Kohlhuber F., Kilger E., Baumann M., Kieser A., Kaiser C., Zeidler R., Scheffer B., Ueffing M., Hammerschmidt W. (1999). Latent Membrane Protein 1 of Epstein-Barr Virus Interacts with JAK3 and Activates STAT Proteins. EMBO J..

[41] Roberts M.L., Cooper N.R. (1998). Activation of a Ras-MAPK-Dependent Pathway by Epstein-Barr Virus Latent Membrane Protein 1 Is Essential for Cellular Transformation. Virology.

[42] Martin G., Roy J., Barat C., Ouellet M., Gilbert C., Tremblay M.J. (2007). Human Immunodeficiency Virus Type 1-Associated CD40 Ligand Transactivates B Lymphocytes and Promotes Infection of CD4+ T Cells. J. Virol..

[43] Imbeault M., Ouellet M., Giguère K., Bertin J., Bélanger D., Martin G., Tremblay M.J. (2011). Acquisition of Host-Derived CD40L by HIV-1 IN VIVO and Its Functional Consequences in the B-Cell Compartment. J. Virol..

[44] Aldinucci D., Gloghini A., Pinto A., Colombatti A., Carbone A. (2012). The Role of CD40/CD40L and Interferon Regulatory Factor 4 in Hodgkin Lymphoma Microenvironment. Leuk. Lymphoma.

[45] Miller C.L., Burkhardt A.L., Lee J.H., Stealey B., Longnecker R., Bolen J.B., Kieff E. (1995). Integral Membrane Protein 2 of Epstein—Barr Virus Regulates Reactivation from Latency through Dominant Negative Effects on Protein-Tyrosine Kinases. Immunity.

[46] Caldwell R.G., Wilson J.B., Anderson S.J., Longnecker R. (1998). Epstein-Barr Virus LMP2A Drives B Cell Development and Survival in the Absence of Normal B Cell Receptor Signals. Immunity.

[47] Green M.R., Rodig S., Juszczynski P., Ouyang J., Sinha P., O’Donnell E., Neuberg D., Shipp M.A. (2012). Constitutive AP-1 Activity and EBV Infection Induce PD-L1 in Hodgkin Lymphomas and Posttransplant Lymphoproliferative Disorders: Implications for Targeted Therapy. Clin. Cancer Res..

[48] Frisan T., Sjöberg J., Dolcetti R., Boiocchi M., De Re V., Carbone A., Brautbar C., Battat S., Biberfeld P., Eckman M. (1995). Local Suppression of Epstein-Barr Virus (EBV)-Specific Cytotoxicity in Biopsies of EBV-Positive Hodgkin’s Disease. Blood.

[49] Chapman A.L., Rickinson A.B., Thomas W.A., Jarrett R.F., Crocker J., Lee S.P. (2001). Epstein-Barr Virus-Specific Cytotoxic T Lymphocyte Responses in the Blood and Tumor Site of Hodgkin’s Disease Patients: Implications for a T-Cell-Based Therapy. Cancer Res..

[50] Marshall N.A., Christie L.E., Munro L.R., Culligan D.J., Johnston P.W., Barker R.N., Vickers M.A. (2004). Immunosuppressive Regulatory T Cells Are Abundant in the Reactive Lymphocytes of Hodgkin Lymphoma. Blood.

[51] Álvaro T., Lejeune M., Salvadó M.T., Bosch R., García J.F., Jaén J., Banham A.H., Roncador G., Montalbán C., Piris M.A. (2005). Outcome in Hodgkin’s Lymphoma Can Be Predicted from the Presence of Accompanying Cytotoxic and Regulatory T Cells. Clin. Cancer Res..

[52] Baráth S., Aleksza M., Keresztes K., Tóth J., Sipka S., Szegedi G., Illés A. (2006). Immunoregulatory T Cells in the Peripheral Blood of Patients with Hodgkin’s Lymphoma. Acta Haematol..

[53] Baumforth K.R.N., Birgersdotter A., Reynolds G.M., Wei W., Kapatai G., Flavell J.R., Kalk E., Piper K., Lee S., Machado L. (2008). Expression of the Epstein-Barr Virus-Encoded Epstein-Barr Virus Nuclear Antigen 1 in Hodgkin’s Lymphoma Cells Mediates Up-Regulation of CCL20 and the Migration of Regulatory T Cells. Am. J. Pathol..

[54] Marshall N.A., Culligan D.J., Tighe J., Johnston P.W., Barker R.N., Vickers M.A. (2007). The Relationships between Epstein-Barr Virus Latent Membrane Protein 1 and Regulatory T Cells in Hodgkin’s Lymphoma. Exp. Hematol..

[55] Kamper P., Bendix K., Hamilton-Dutoit S., Honoré B., Nyengaard J.R., d’Amore F. (2011). Tumor-Infiltrating Macrophages Correlate with Adverse Prognosis and Epstein-Barr Virus Status in Classical Hodgkin’s Lymphoma. Haematologica.

[56] Tan K.L., Scott D.W., Hong F., Kahl B.S., Fisher R.I., Bartlett N.L., Advani R.H., Buckstein R., Rimsza L.M., Connors J.M. (2012). Tumor-Associated Macrophages Predict Inferior Outcomes in Classic Hodgkin Lymphoma: A Correlative Study from the E2496 Intergroup Trial. Blood.

[57] Mills C.D. (2015). Anatomy of a Discovery: M1 and M2 Macrophages. Front. Immunol..

[58] Barros M.H.M., Hauck F., Dreyer J.H., Kempkes B., Niedobitek G. (2013). Macrophage Polarisation: An Immunohistochemical Approach for Identifying M1 and M2 Macrophages. PLoS ONE.

[59] Hartmann S., Jakobus C., Rengstl B., Döring C., Newrzela S., Brodt H.-R., Wolf T., Hansmann M.-L. (2013). Spindle-Shaped CD163+ Rosetting Macrophages Replace CD4+ T-Cells in HIV-Related Classical Hodgkin Lymphoma. Mod. Pathol..

[60] Cassol E., Cassetta L., Rizzi C., Alfano M., Poli G. (2009). M1 and M2a Polarization of Human Monocyte-Derived Macrophages Inhibits HIV-1 Replication by Distinct Mechanisms. J. Immunol..

[61] Thompson L.D.R., Fisher S.I., Chu W.S., Nelson A., Abbondanzo S.L. (2004). HIV-Associated Hodgkin Lymphoma: A Clinicopathologic and Immunophenotypic Study of 45 Cases. Am. J. Clin. Pathol..

[62] Koulis A., Trivedi P., Ibrahim H., Bower M., Naresh K.N. (2014). The Role of the Microenvironment in Human Immunodeficiency Virus-Associated Classical Hodgkin Lymphoma. Histopathology.

[63] Scala G., Ruocco M.R., Ambrosino C., Mallardo M., Giordano V., Baldassarre F., Dragonetti E., Quinto I., Venuta S. (1994). The Expression of the Interleukin 6 Gene Is Induced by the Human Immunodeficiency Virus 1 TAT Protein. J. Exp. Med..

[64] Blazevic V., Heino M., Lagerstedt A., Ranki A., Krohn K.J. (1996). Interleukin-10 Gene Expression Induced by HIV-1 Tat and Rev in the Cells of HIV-1 Infected Individuals. J. Acquir. Immune Defic. Syndr. Hum. Retrovirol..

[65] Vendrame E., Hussain S.K., Breen E.C., Magpantay L.I., Widney D.P., Jacobson L.P., Variakojis D., Knowlton E.R., Bream J.H., Ambinder R.F. (2014). Serum Levels of Cytokines and Biomarkers for Inflammation and Immune Activation, and HIV-Associated Non-Hodgkin B-Cell Lymphoma Risk. Cancer Epidemiol. Biomark. Prev..

[66] Muramatsu M., Kinoshita K., Fagarasan S., Yamada S., Shinkai Y., Honjo T. (2000). Class Switch Recombination and Hypermutation Require Activation-Induced Cytidine Deaminase (AID), a Potential RNA Editing Enzyme. Cell.

[67] Robbiani D.F., Bunting S., Feldhahn N., Bothmer A., Camps J., Deroubaix S., McBride K.M., Klein I.A., Stone G., Eisenreich T.R. (2009). AID Produces DNA Double-Strand Breaks in Non-Ig Genes and Mature B Cell Lymphomas with Reciprocal Chromosome Translocations. Mol. Cell.

[68] Swerdlow S.H., Campo E., Pileri S.A., Harris N.L., Stein H., Siebert R., Advani R., Ghielmini M., Salles G.A., Zelenetz A.D. (2016). The 2016 Revision of the World Health Organization Classification of Lymphoid Neoplasms. Blood.

[69] Hentrich M., Maretta L., Chow K.U., Bogner J.R., Schürmann D., Neuhoff P., Jäger H., Reichelt D., Vogel M., Ruhnke M. (2006). Highly Active Antiretroviral Therapy (HAART) Improves Survival in HIV-Associated Hodgkin’s Disease: Results of a Multicenter Study. Ann. Oncol..

[70] Corti M., Villafañe M., Minue G., Campitelli A., Narbaitz M., Gilardi L. (2015). Clinical Features of AIDS Patients with Hodgkin’s Lymphoma with Isolated Bone Marrow Involvement: Report of 12 Cases at a Single Institution. Cancer Biol. Med..

[71] Muthukrishnan S., Amudhan A., Rajendran S. (2018). Primary Hodgkin’s Lymphoma of Liver in HIV—A Case Report and Review of Literature. AME Case Rep..

[72] Spina M., Gabarre J., Rossi G., Fasan M., Schiantarelli C., Nigra E., Mena M., Antinori A., Ammassari A., Talamini R. (2002). Stanford V Regimen and Concomitant HAART in 59 Patients with Hodgkin Disease and HIV Infection. Blood.

[73] Hartmann P., Rehwald U., Salzberger B., Franzen C., Sieber M., Wöhrmann A., Diehl V. (2003). BEACOPP Therapeutic Regimen for Patients with Hodgkin’s Disease and HIV Infection. Ann. Oncol..

[74] Hentrich M., Berger M., Wyen C., Siehl J., Rockstroh J.K., Müller M., Fätkenheuer G., Seidel E., Nickelsen M., Wolf T. (2012). Stage-Adapted Treatment of HIV-Associated Hodgkin Lymphoma: Results of a Prospective Multicenter Study. J. Clin. Oncol..

[75] Castillo J.J., Bower M., Brühlmann J., Novak U., Furrer H., Tanaka P.Y., Besson C., Montoto S., Cwynarski K., Abramson J.S. (2015). Prognostic Factors for Advanced-Stage Human Immunodeficiency Virus-Associated Classical Hodgkin Lymphoma Treated with Doxorubicin, Bleomycin, Vinblastine, and Dacarbazine plus Combined Antiretroviral Therapy: A Multi-Institutional Retrospective Study. Cancer.

[76] Yotsumoto M., Ito Y., Hagiwara S., Terui Y., Nagai H., Ota Y., Ajisawa A., Uehira T., Tanuma J., Ohyashiki K. (2018). HIV Positivity May Not Have a Negative Impact on Survival in Epstein-Barr Virus-Positive Hodgkin Lymphoma: A Japanese Nationwide Retrospective Survey. Oncol. Lett..

[77] Lawal I.O., Ankrah A.O., Popoola G.O., Nyakale N.E., Boshomane T.G., Reyneke F., Lengana T., Vorster M., Sathekge M.M. (2018). 18F-FDG-PET Metabolic Metrics and International Prognostic Score for Risk Assessment in HIV-Infected Patients with Hodgkin Lymphoma. Nucl. Med. Commun..

[78] Okosun J., Warbey V., Shaw K., Montoto S., Fields P., Marcus R., Virchis A., McNamara C., Bower M., Cwynarski K. (2012). Interim Fluoro-2-Deoxy-D-Glucose-PET Predicts Response and Progression-Free Survival in Patients with Hodgkin Lymphoma and HIV Infection. AIDS.

[79] Danilov A.V., Li H., Press O.W., Shapira I., Swinnen L.J., Noy A., Reid E., Smith S.M., Friedberg J.W. (2017). Feasibility of Interim Positron Emission Tomography (PET)-Adapted Therapy in HIV-Positive Patients with Advanced Hodgkin Lymphoma (HL): A Sub-Analysis of SWOG S0816 Phase 2 Trial. Leuk. Lymphoma.

[80] Rubinstein P.G., Moore P.C., Rudek M.A., Henry D.H., Ramos J.C., Ratner L., Reid E., Sharon E., Noy A., AIDS Malignancy Consortium (AMC) (2018). Brentuximab Vedotin with AVD Shows Safety, in the Absence of Strong CYP3A4 Inhibitors, in Newly Diagnosed HIV-Associated Hodgkin Lymphoma. AIDS.

[81] Krishnan A., Molina A., Zaia J., Smith D., Vasquez D., Kogut N., Falk P.M., Rosenthal J., Alvarnas J., Forman S.J. (2005). Durable Remissions with Autologous Stem Cell Transplantation for High-Risk HIV-Associated Lymphomas. Blood.

[82] Spitzer T.R., Ambinder R.F., Lee J.Y., Kaplan L.D., Wachsman W., Straus D.J., Aboulafia D.M., Scadden D.T. (2008). Dose-Reduced Busulfan, Cyclophosphamide, and Autologous Stem Cell Transplantation for Human Immunodeficiency Virus-Associated Lymphoma: AIDS Malignancy Consortium Study 020. Biol. Blood Marrow Transplant..

[83] Re A., Michieli M., Casari S., Allione B., Cattaneo C., Rupolo M., Spina M., Manuele R., Vaccher E., Mazzucato M. (2009). High-Dose Therapy and Autologous Peripheral Blood Stem Cell Transplantation as Salvage Treatment for AIDS-Related Lymphoma: Long-Term Results of the Italian Cooperative Group on AIDS and Tumors (GICAT) Study with Analysis of Prognostic Factors. Blood.

[84] Durand C.M., Capoferri A.A., Redd A.D., Zahurak M., Rosenbloom D.I.S., Cash A., Avery R.K., Bolaños-Meade J., Bollard C.M., Bullen C.K. (2020). Allogeneic Bone Marrow Transplantation with Post-Transplant Cyclophosphamide for Patients with HIV and Haematological Malignancies: A Feasibility Study. Lancet HIV.

[85] Chang E., Rivero G., Patel N.R., Chiao E.Y., Lai S., Bajaj K., Mbue J.E., Yellapragada S.V. (2018). HIV-Related Refractory Hodgkin Lymphoma: A Case Report of Complete Response to Nivolumab. Clin. Lymphoma Myeloma Leuk..

[86] Sandoval-Sus J.D., Mogollon-Duffo F., Patel A., Visweshwar N., Laber D.A., Kim R., Jagal M.V. (2017). Nivolumab as Salvage Treatment in a Patient with HIV-Related Relapsed/Refractory Hodgkin Lymphoma and Liver Failure with Encephalopathy. J. Immunother. Cancer.

[87] Cullen M., Steven N., Billingham L., Gaunt C., Hastings M., Simmonds P., Stuart N., Rea D., Bower M., Fernando I. (2005). Antibacterial Prophylaxis after Chemotherapy for Solid Tumors and Lymphomas. N. Engl. J. Med..

[88] Re A., Cattaneo C., Montoto S. (2020). Treatment Management of Haematological Malignancies in People Living with HIV. Lancet Haematol..

[89] Powles T., Imami N., Nelson M., Gazzard B.G., Bower M. (2002). Effects of Combination Chemotherapy and Highly Active Antiretroviral Therapy on Immune Parameters in HIV-1 Associated Lymphoma. AIDS.

[90] Esdaile B., Davis M., Portsmouth S., Sarker D., Nelson M., Gazzard B., Bower M. (2002). The Immunological Effects of Concomitant Highly Active Antiretroviral Therapy and Liposomal Anthracycline Treatment of HIV-1-Associated Kaposi’s Sarcoma. AIDS.

[91] Alfa-Wali M., Allen-Mersh T., Antoniou A., Tait D., Newsom-Davis T., Gazzard B., Nelson M., Bower M. (2012). Chemoradiotherapy for Anal Cancer in HIV Patients Causes Prolonged CD4 Cell Count Suppression. Ann. Oncol..

[92] Uldrick T.S., Little R.F. (2015). How I Treat Classical Hodgkin Lymphoma in Patients Infected with Human Immunodeficiency Virus. Blood.

[93] Little R.F., Dunleavy K. (2013). Update on the Treatment of HIV-Associated Hematologic Malignancies. Hematol. Am. Soc. Hematol. Educ. Program..

[94] Navarro J.-T., Ribera J.-M., Oriol A., Romeu J., Sirera G., Mate J.-L., Batlle M., Xicoy B., Grau J., Millá F. (2002). Favorable Impact of Virological Response to Highly Active Antiretroviral Therapy on Survival in Patients with AIDS-Related Lymphoma. Leuk. Lymphoma.

[95] Weiss R., Mitrou P., Arasteh K., Schuermann D., Hentrich M., Duehrsen U., Sudeck H., Schmidt-Wolf I.G.H., Anagnostopoulos I., Huhn D. (2006). Acquired Immunodeficiency Syndrome-Related Lymphoma: Simultaneous Treatment with Combined Cyclophosphamide, Doxorubicin, Vincristine, and Prednisone Chemotherapy and Highly Active Antiretroviral Therapy Is Safe and Improves Survival--Results of the German Multicenter Trial. Cancer.

[96] Mounier N., Spina M., Gabarre J., Raphael M., Rizzardini G., Golfier J.-B., Vaccher E., Carbone A., Coiffier B., Chichino G. (2006). AIDS-Related Non-Hodgkin Lymphoma: Final Analysis of 485 Patients Treated with Risk-Adapted Intensive Chemotherapy. Blood.

[97] Gopal S., Patel M.R., Yanik E.L., Cole S.R., Achenbach C.J., Napravnik S., Burkholder G.A., Reid E.G., Rodriguez B., Deeks S.G. (2013). Association of Early HIV Viremia with Mortality after HIV-Associated Lymphoma. AIDS.

[98] Vaccher E., Spina M., di Gennaro G., Talamini R., Nasti G., Schioppa O., Vultaggio G., Tirelli U. (2001). Concomitant Cyclophosphamide, Doxorubicin, Vincristine, and Prednisone Chemotherapy plus Highly Active Antiretroviral Therapy in Patients with Human Immunodeficiency Virus-Related, Non-Hodgkin Lymphoma. Cancer.

[99] Antinori A., Cingolani A., Alba L., Ammassari A., Serraino D., Ciancio B.C., Palmieri F., De Luca A., Larocca L.M., Ruco L. (2001). Better Response to Chemotherapy and Prolonged Survival in AIDS-Related Lymphomas Responding to Highly Active Antiretroviral Therapy. AIDS.

[100] El-Sadr W.M., Lundgren J.D., Neaton J.D., Gordin F., Abrams D., Arduino R.C., Babiker A., Burman W., Clumeck N., Strategies for Management of Antiretroviral Therapy (SMART) Study Group (2006). CD4+ Count-Guided Interruption of Antiretroviral Treatment. N. Engl. J. Med..

[101] Torres H.A., Mulanovich V. (2014). Management of HIV Infection in Patients with Cancer Receiving Chemotherapy. Clin. Infect. Dis..

[102] Department of Health and Human Services Panel on Antiretroviral Guidelines for Adults and Adolescents. Guidelines for the Use of Antiretroviral Agents in HIV-1-Infected Adults and Adolescents.

[103] King J.R., Wynn H., Brundage R., Acosta E.P. (2004). Pharmacokinetic Enhancement of Protease Inhibitor Therapy. Clin. Pharmacokinet..

[104] Shah B.M., Schafer J.J., Priano J., Squires K.E. (2013). Cobicistat: A New Boost for the Treatment of Human Immunodeficiency Virus Infection. Pharmacotherapy.

[105] Sombogaard F., Franssen E.J.F., Terpstra W.E., Kerver E.D., van den Berk G.E.L., Crul M. (2018). Outcome Effects of Antiretroviral Drug Combinations in HIV-Positive Patients with Chemotherapy for Lymphoma: A Retrospective Analysis. Int. J. Clin. Pharm..

[106] Focà E., Cavaglià G., Rusconi S., Cascavilla A., Cenderello G., Re A., Casari S., van den Bogaart L., Zinzani P.L., Caracciolo D. (2018). Survival in HIV-Infected Patients with Lymphoma According to the Choice of Antiretroviral Treatment: An Observational Multicentre Study. HIV Med..

[107] Torres H.A., Rallapalli V., Saxena A., Granwehr B.P., Viola G.M., Ariza-Heredia E., Adachi J.A., Chemaly R.F., Marfatia R., Jiang Y. (2014). Efficacy and Safety of Antiretrovirals in HIV-Infected Patients with Cancer. Clin. Microbiol. Infect..

[108] Bower M., Powles T., Stebbing J., Thirlwell C. (2005). Potential Antiretroviral Drug Interactions with Cyclophosphamide, Doxorubicin, and Etoposide. J. Clin. Oncol..

[109] Levêque D., Santucci R., Pavillet J., Herbrecht R., Bergerat J.P. (2009). Paralytic Ileus Possibly Associated with Interaction between Ritonavir/Lopinavir and Vincristine. Pharm. World Sci. PWS.

[110] Liedtke M.D., Tomlin C.R., Lockhart S.M., Miller M.M., Rathbun R.C. (2014). Long-Term Efficacy and Safety of Raltegravir in the Management of HIV Infection. Infect. Drug Resist..

[111] Cottrell M.L., Hadzic T., Kashuba A.D.M. (2013). Clinical Pharmacokinetic, Pharmacodynamic and Drug-Interaction Profile of the Integrase Inhibitor Dolutegravir. Clin. Pharmacokinet..

[112] Zeuli J., Rizza S., Bhatia R., Temesgen Z. (2019). Bictegravir, a Novel Integrase Inhibitor for the Treatment of HIV Infection. Drugs Today Barc..

[113] Moltó J., Rajoli R., Back D., Valle M., Miranda C., Owen A., Clotet B., Siccardi M. (2017). Use of a Physiologically Based Pharmacokinetic Model to Simulate Drug-Drug Interactions between Antineoplastic and Antiretroviral Drugs. J. Antimicrob. Chemother..

[114] Sharma M., Saravolatz L.D. (2013). Rilpivirine: A New Non-Nucleoside Reverse Transcriptase Inhibitor. J. Antimicrob. Chemother..

[115] Khalilieh S., Yee K.L., Sanchez R., Stoch S.A., Wenning L., Iwamoto M. (2020). Clinical Pharmacokinetics of the Novel HIV-1 Non-Nucleoside Reverse Transcriptase Inhibitor Doravirine: An Assessment of the Effect of Patient Characteristics and Drug-Drug Interactions. Clin. Drug Investig..

[116] Scherzer R., Estrella M., Li Y., Choi A.I., Deeks S.G., Grunfeld C., Shlipak M.G. (2012). Association of Tenofovir Exposure with Kidney Disease Risk in HIV Infection. AIDS.

[117] James C.W., Szabo S., Kahal D., Goldstein N.D. (2020). The Effect of Multivitamins and Polyvalent Cations on Virologic Suppression with Integrase Strand Transfer Inhibitors. AIDS.

[118] Rock A.E., DeMarais P.L., Vergara-Rodriguez P.T., Max B.E. (2020). HIV-1 Virologic Rebound Due to Coadministration of Divalent Cations and Bictegravir. Infect. Dis. Ther..

[119] Liverpool HIV Interactions. https://www.hiv-druginteractions.org/.

[120] Immunodeficiency Clinic. https://hivclinic.ca/main/drugs_interact.html.

[121] Rubinstein P.G., Aboulafia D.M., Zloza A. (2014). Malignancies in HIV/AIDS: From Epidemiology to Therapeutic Challenges. AIDS.

[122] Rudek M.A., Flexner C., Ambinder R.F. (2011). Use of Antineoplastic Agents in Patients with Cancer Who Have HIV/AIDS. Lancet Oncol..

